# MgO Nanoparticles Obtained from an Innovative and Sustainable Route and Their Applications in Cancer Therapy

**DOI:** 10.3390/nano13222975

**Published:** 2023-11-19

**Authors:** Valeria Daniele, Anna Rita Volpe, Patrizia Cesare, Giuliana Taglieri

**Affiliations:** 1 Department of Industrial and Information Engineering and Economics, University of L’Aquila, Piazzale E. Pontieri 1, Monteluco di Roio, Roio Poggio, 67100 L’Aquila, Italy; 2 Department of Life, Health and Environmental Sciences, University of L’Aquila, Edificio Renato Ricamo, Via Vetoio, Coppito, 67100 L’Aquila, Italy; annarita.volpe@univaq.it (A.R.V.); patrizia.cesare@univaq.it (P.C.)

**Keywords:** MgO nanoparticles, scalable and time-energy synthesis, ion exchange process, XRD, HRTEM, therapeutic applications, MgO against cancer growth, growth curves of melanoma cells

## Abstract

This paper aimed to evaluate the biological damages towards diseased cells caused by the use of MgO nanoparticles (NPs). The NPs are produced by a calcination process of a precursor, which is an aqueous suspension of nanostructured Mg(OH)_2_, in turn synthesized following our original, time-energy saving and scalable method able to guarantee short times, high yield of production (up to almost 10 kg/week of NPs), low environmental impact and low energy demand. The MgO NPs, in the form of dry powders, are organized as a network of intercrystallite channels, in turn constituted by monodispersed and roughly spherical NPs < 10 nm, preserving the original pseudo hexagonal-platelet morphology of the precursor. The produced MgO powders are diluted in a PBS solution to obtain different MgO suspension concentrations that are subsequently put in contact, for 3 days, with melanoma and healthy cells. The viable count, made at 24, 48 and 72 h from the beginning of the test, reveals a good cytotoxic activity of the NPs, already at low MgO concentrations. This is particularly marked after 72 h, showing a clear reduction in cellular proliferation in a MgO-concentration-dependent manner. Finally, the results obtained on human skin fibroblasts revealed that the use MgO NPs did not alter at all both the vitality and proliferation of healthy cells.

## 1. Introduction

One of the major diseases currently afflicting the population worldwide is cancer, in which a group of cells reveal an uncontrolled growth into the body that leads, sometimes, to the formation of metastasis [1,2]. The conventional approaches used to treat cancer are chemotherapy, radiation and surgery; nevertheless, these procedures often present severe limitations since they can affect both diseased and healthy cells in the body.

So, the possibility of employing metal oxide nanoparticles (NPs) in place of harmful techniques has become the current intriguing challenge, allowing for the establishment of a new criterion for the development of NPs in the medical field, where extensive applications are required [1,2,3,4]. This is due to the anticancer activity of the NPs depending on their intrinsic features such as antioxidant action, as well as to their ability to interact with proteins, nucleic acids and lipids, both inside and outside the cell.

In particular, the potential cytotoxicity of NPs against cancer cells is related to oxidative stress stimulated by reactive oxygen species (ROS), leading to an apoptotic process and inducing significant cell structure damage to membrane lipids, membrane proteins and to the nuclear membrane [2,3,5,6,7,8,9,10,11].

Metal oxide NPs are also able to produce free radicals that kill cancer cells if stimulated by an external radiation source, such as hyperthermia, in response to the application of infrared rays or magnetic fields. In particular, the NPs acting as co-adjuvant agents can increase the cell killing effect of ionizing radiation during the radiotherapy process, specifically on cancer cells [1].

Moreover, these NPs can allow a site-specific release of therapeutic drugs targeted towards cancer cells, guaranteeing an improvement in their biodistribution, a longer shelf life and an administration of both hydrophilic and hydrophobic substances through oral, nasal, intraocular and parenteral routes [12,13]. Due to their ability to overpower cellular strategies blocking foreign bodies, the NPs can make it easy for the drugs to target the cancer cells, thus decreasing their dangerous effects on healthy ones [14].

Metal oxide NPs are experimentally used to directly kill cancer cells both in vitro and in vivo [5] and nowadays, among all of them, magnesium oxide nanoparticles (MgO NPs) have been established at the forefront of nanomedical research, thanks to their considerable potential for treating a sore stomach, bone regeneration and as an antibacterial agent [15,16,17]. This is due to their interesting properties in terms of stability, crystallinity, absorptivity, large surface area and reactivity. Moreover, a not-negligible issue is that MgO NPs, differently from other kind of metal oxide (such as ZnO and TiO_2_), can be considered biocompatible with the human organism, making these NPs even more promising in terms of their antibacterial activity [18]. More recently, MgO NPs have also been applied in cancer therapy, such as in nano-cryosurgery, hyperthermia and as chemotherapeutic agents for the rapid detection and identification of all cancer types [19,20,21].

As reported in the literature, several methods for synthesizing MgO NPs with high quality, improved monodispersity and crystallinity are available; they include precipitation, microwave-induced combustion, hydrothermal processes, flame spray pyrolysis, aerosol combustion, chemical vapor deposition, flame metal combustion and sol–gel [16,22,23,24,25,26,27]. Nevertheless, these methods are often characterized by limitations due to high temperature/pressure, expensive or sophisticated apparatuses, long synthesis times and a low yield of NP production. In addition, green synthesis procedures based on the use of biological extracts have been proposed, allowing for obtaining MgO NPs in a cost-effective way [28,29,30]. These biological processes, although environmentally friendly and less hazardous than physicochemical ones, pose concerns regarding the scale-up in NP production, in relation to the complexity of the biological extracts created during the synthesis, a barrier to the elucidation of the reactions and their mechanisms [28]. More recently, the solution-phase methods have gained greater attention, demonstrating the possibility of producing MgO NPs starting from the precipitation of a precursor that is magnesium hydroxide (Mg(OH)_2_). In this task, the precursor features play a fundamental role as they affect the process of topochemical decomposition, influencing the size and morphology of the produced MgO NPs [31,32]. However, even these methods present drawbacks mainly related to high temperatures and multistep procedures (purifications/washings), resulting in long synthesis times and a low yield of production, which is a crucial limitation for the large-scale application of the MgO NPs.

The possibility, provided by our innovative and sustainable ion exchange synthetic route, to produce in only 15 min up to almost 10 kg/week of pure and crystalline Mg(OH)_2_ NPs, useful as precursors to MgO ones, can constitute an important goal in defining the starting point for the scale-up in NP production to the market requests, particularly in the medical field where large amounts of NPs are necessary. The process is a time–energy saving and eco-friendly synthetic route that works in a single step, at an ambient temperature/pressure, with renewable reagents, low energy consumption and without any toxic waste [33], guaranteeing a drastic reduction in the synthesis times and a scale up in the NP production.

Profiting from the productivity of our cyclic route, the aim of this paper was to evaluate the cytotoxicity of the MgO NPs towards cancer formation and growth by means of in vitro tests. The tests were aimed at establishing the biological damages caused by the NPs in terms of their released of toxic chemicals and ability to kill diseased cells by means of inhibition of cell metabolic pathways.

In particular, starting from the synthesis of the precursor—Mg(OH)_2_ NPs—and following a calcination process, pure and crystalline MgO NPs were obtained. Both Mg(OH)_2_ and MgO NPs were characterized from structural and morphological points of view, by means of an X-ray diffraction (profile fitting and Rietveld refinement) technique, transmission electron microscopy (TEM/HRTEM) and surface area measurements (BET).

The MgO NPs, in the form of dry powders, were dispersed in a phosphate buffered saline (PBS) solution, giving rise to different MgO suspension concentrations that were injected into a culture of melanoma cells. After 24, 48 and 72 h of treatment that were performed considering different MgO concentrations, a viable count was carried out and the number of live cells at the different incubation times was established. Then, the growth curves, expressed as a function of the cell culture time and of the MgO concentration, were determined. Finally, the evaluation of the influence of MgO NPs on human skin fibroblasts (HS27) was investigated as well.

## 2. Materials

The materials employed to synthesize the precursor, that were Mg(OH)_2_ NPs, were magnesium chloride (MgCl_2_), with a purity > 98%, supplied by Merck (Union County, NJ, USA), and an ion-exchange resin Dowex Monosphere 550A (Lennthech, Delft, The Netherlands), in form of translucent spherical beads characterized by a particle size equal to 590 ± 50 µm, supplied by Sigma Aldrich (St. Louis, MO, USA).

### 2.1. Synthesis of MgO NPs Obtained Starting from Mg(OH)_2_ Precursor

The synthesis of the precursor—Mg(OH)_2_ NPs in form of an aqueous suspension—was performed by means of our innovative and sustainable procedure, already patented for the production of different metal oxide/hydroxide NPs [34,35].

In particular, the sustainability of the process lies in the possibility to work in water, at room temperature and ambient pressure, with low environmental impact, low energy consumption and without the production of any toxic waste. Briefly, a 1 M MgCl_2_ aqueous solution was put in contact, for only 15 min and under moderate stirring, with a proper amount of anionic resin, working at room temperature (T = 25 °C). After a few seconds, the precipitation of solid Mg(OH)_2_ occurred and, at the end of the synthesis, the Mg(OH)_2_ aqueous suspension (from here called **MH**) was separated from the resin by means of a sieving procedure [33]. During the stirring operation, the kinetics of the ion exchange process were determined by taking homogeneous samples from the suspension, at different reaction times with the resin (t = 0, 5, 15, 30, 60, 75, 180, 300, 600 and 900 s, respectively). By using an ion-sensitive electrode (Metrohm, Herisau, Switzerland), the variation in the chloride concentration, from the beginning to the end of the synthesis, was measured. From the obtained results, the process appeared characterized by very fast kinetics in terms of **MH** NP production, giving rise to a reduction in the chloride content of about 90% in the first 30 s, with a residual chloride content at the end of the synthesis of (13.3 ± 0.1) mg/L. In addition, the possibility of using renewable reagents allowed for the scale up of the NP production of up to almost 10 kg/week.

Firstly, the **MH** precursor was dried at 110 °C for 24 h in a laboratory oven (Memmert TV30b, Enco, Venezia, Italy) to reduce the volume of the sample. Subsequently, in order to obtain the MgO sample (**MgO**), the dried powders of the precursor were grounded and calcinated at 500 °C for 4 h, by using a Lenton furnace (Lenton thermal designs LTD, London, UK) with a heating rate of 10 °C/min. In particular, the optimal temperature range to be used for the calcination process was established according to the methodology proposed in our previous paper [32].

### 2.2. Characterization of the Produced MgO NPs

The **MH** precursor, as well as the obtained **MgO** powders, were characterized by XRD, TEM/HRTEM and BET measurements. Phase purity and crystallinity of **MH** and **MgO** samples were analysed by means of XRD spectra, recorded on a PANalytical X’PertPRO apparatus (Almelo, The Netherlands) using CuK_α_ radiation, with a step scan, covering the angular range 2θ from 10° to 90°, and a step size 2θ = 0.026°. The experimental diffraction patterns were elaborated by a Profile Fit Software Version 4.9 (HighScorePlus software package, PANalytical, Cedar Park, TX, USA), and crystalline phases were attributed by the international ICDD and ICSD reference databases. In addition, XRD peak broadening analysis was carried out to evaluate the average crystallite size, D_hkl_, through the Debye–Scherrer formula [36]. The particle morphology was investigated by means of transmission and high-resolution electron microscopy (TEM (Thermo Fischer Scientific Brno s.r.o., Brno, Czech Republic), Philips CM100 and HRTEM (FEI Company, Hillsboro, OR, USA), TECNAI G2 TF30 STEM, respectively), according to standard procedures, while the particle dimensions were calculated by using ImageJ software (Java 1.6.0_20).

Finally, for the surface area measurements, nitrogen adsorption analysis was carried out at 77 K, using a Quantachrome Nova system utilizing Brunauer–Emmett–Teller (BET). Both **MH** and **MgO** samples (approximately 0.2 g dry powders) were first outgassed for about 2 h at 150 °C, then for about 16 h at 250 °C, (5·10^−3^ Torr). The pore size distribution was determined from the desorption branch of the isotherms using the BJH (Barett–Joyner–Halenda) method.

### 2.3. In Vitro Tests to Evaluate the MgO NPs’ Efficacy as Toxicological Agents against Cancer Cells

First of all, a stock suspension, containing the produced **MgO** powders dispersed in a phosphate buffered saline (PBS) solution, was prepared. This suspension, characterized by a **MgO** concentration equal to 80 mg/mL—and from here called **MgO_80_**—was sonicated for 15 min by using an ultrasound tip sonicator (Vibra-Cell™ Ultrasonic VCX 400, Tecnochimica, Grottazzolina, Italy).

Subsequently, to evaluate the effect of MgO NPs towards the proliferation and viability of both cancer and healthy cells, melanoma cells (Bmel) and human skin fibroblasts (HS27) were considered. The trypan blue dye exclusion test (TBDET) was used to determine the viability of the considered cells, whose proliferations were assessed by exposing the cells to different **MgO** suspension concentrations, for various times.

These cells were grown in Dulbecco’s modified Eagle medium, supplemented with 10% fetal bovine serum, 2 mM L-glutamine, 100 IU/mL penicillin and 100 µg/mL streptomycin; the whole system was maintained at a fixed temperature of 37 °C, working in a humidified atmosphere with 5% CO_2_. The medium was replaced every 3 days, and the cells were detached and sub-cultured when ≊90% confluence was reached.

A total of 70,000 cells for Bmel and 100,000 cells for HS27 (in 1.5 mL of culture medium/well) were seeded into cell culture dishes (growth area 10 cm^2^) and after 24 h the cells were exposed to the **MgO** NPs.

In particular, by dilution of the initial **MgO_80_**, six **MgO** suspensions in PBS were prepared, having **MgO** concentrations equal to 5, 10, 20, 30, 40 and 50 mg/mL, respectively. Each suspension was sonicated for 15 min, and then 15 μL were picked and added to the culture plates until obtaining final **MgO** concentrations of 50, 100, 200, 300, 400 and 500 µg/mL (**MgO_50_**, **MgO_100_**, **MgO_200_**, **MgO_300_**, **MgO_400_** and **MgO_500_**, respectively). These suspensions were put in contact both with melanoma and healthy cells for 3 days.

After 24, 48 and 72 h from the beginning of the treatment, the cell counting was performed by using a Neubauer hemocytometer and a Nikon Eclipse TS 100 (Tokyo, Japan) inverted microscope, equipped with a phase contrast objective. The cell viability was assessed by removing cells from the plates with 0.05% trypsin-0.02% EDTA solution and by combining 20 µL aliquots of this cell suspension with 20 µL of 4% trypan blue dye solution. The dye stained the damaged cells leaving the undamaged ones colorless, while a diffuse cytoplasmic staining underlined the cell death [37].

For each **MgO** suspension concentration, two dishes were counted, and this procedure was repeated two times; the obtained results were compared with those coming from melanoma and healthy cells without the addition of **MgO** NPs.

Finally, by means of a calculator [38,39], the concentration at which a substance exerts half of its maximal inhibitory effect (IC_50_) was determined.

## 3. Results

**Figure 1:**
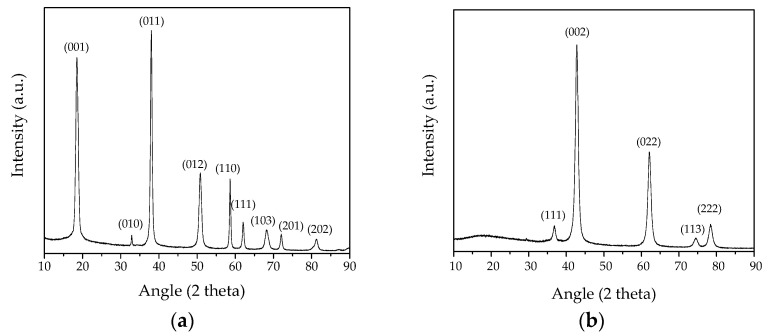
X-ray diffraction pattern of the synthesized nanoparticles. (**a**) The precursor Mg(OH)_2_ (**MH** sample); (**b**) MgO NPs obtained after the calcination process (**MgO** sample).

The synthesized **MH** nanoparticles, dried in an oven at 60 °C, were characterized in terms of crystallinity and phase purity by means of XRD investigation. The hexagonal brucite structure (ICSD #98-008-9823), with cell parameters *a* and *c* calculated by the Rietveld method (Table 1), was recognized and no secondary phases were detected, denoting the phase purity of the obtained crystalline **MH** NPs (Figure 1a). Moreover, the Bragg peaks appeared broadened, underlining the small dimension of the produced crystallites having an average crystallite size <D_hkl_> value of about 14 nm, as calculated by the Debye–Scherrer formula (see Table 1).

The **MH** precursor was then calcinated at 500 °C for 4 h, according to the methodology proposed in [32] and, during this time, a complete decomposition process of Mg(OH)_2_ to MgO occurred, as reported in (1):

Mg(OH)_2_ → MgO + H_2_O (1)

Following the calcination process, pure and crystalline **MgO** NPs were produced, as observed from the XRD spectrum, which revealed that as all the peaks well matched the standard diffraction pattern of MgO (ICSD #98-017-0905), no trace of impurities can be recognized (Figure 1b). Here too, the peaks appear broadened showing a <D_hk_> value of less than 9 nm (Table 1). From the analysis of the D_hkl_ values, no particularly marked differences in widths between the Bragg peaks were denoted, underlining the spherical shape of the produced NPs [40].

**Figure 2:**
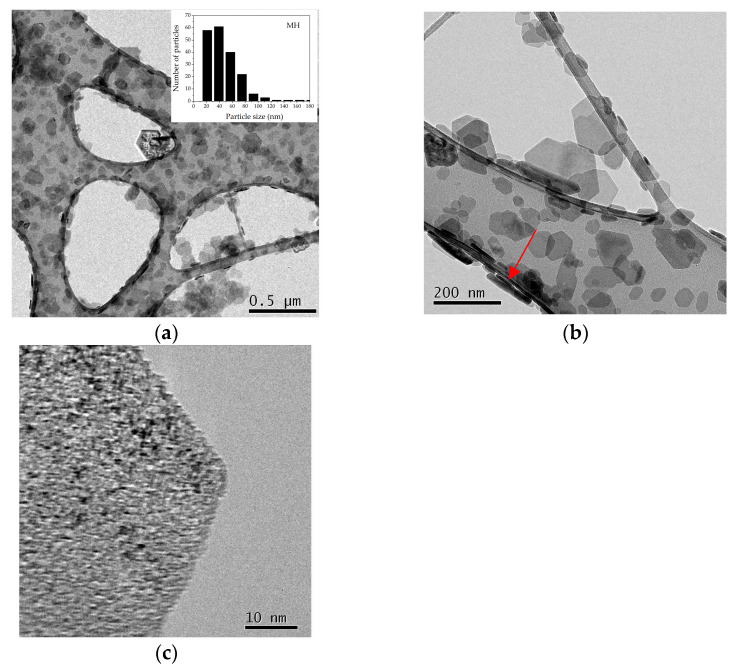
HRTEM images of **MH** nanoparticles acting as a precursor. (**a**,**b**) The presence of pseudo-hexagonal lamellas much lower than 90 nm with thicknesses less than 10 nm can be observed; (**c**) at higher magnification, each lamella was really composed by a self-assembly of primary and homogeneously dispersed nanoparticles < 10 nm.

Morphology and particle dimensions of both **MH** and **MgO** samples were examined by means of TEM and HRTEM investigations. Concerning the **MH** sample, the presence of pseudo-hexagonal lamellas characterized by an average size ranging from 30 to 90 nm and thickness ≤ 10 nm was recognized (see the inset in Figure 2a and the arrow in Figure 2b, respectively). Really, as also observed in our previous works [32,33], at higher magnification each lamella appeared to be composed by a dense and oriented aggregation of primary nanoparticles < 10 nm, homogeneously dispersed and acting as nanosized precursors (Figure 2c).

**Figure 3:**
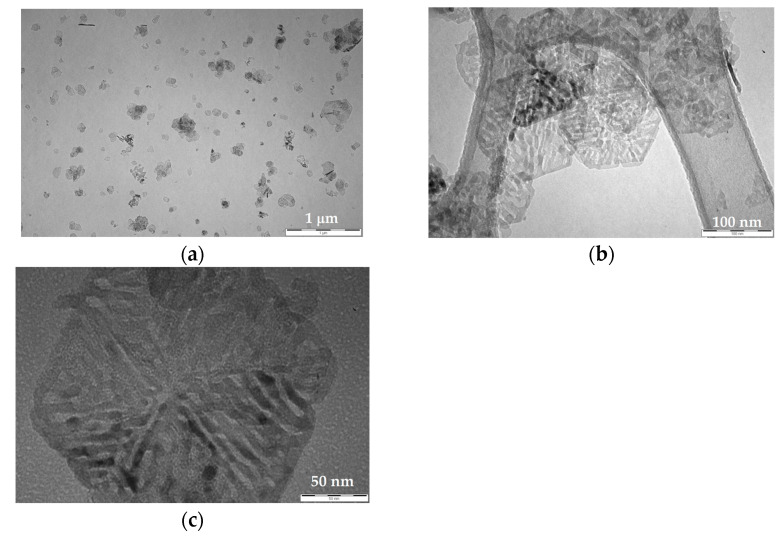
TEM images of the produced **MgO** NPs obtained starting from the calcination process of the precursor. (**a**,**b**) Following the pseudomorphic decomposition of **MH**, the formation of a network of intercrystallite channels of **MgO** NPs can be observed; (**c**) each pseudo-hexagonal lamella is composed by an aggregation of monodispersed and roughly spherical **MgO** NPs, less than 10 nm.

As soon as the decomposition process of the precursor occurred, a pseudomorphic transformation of **MH** can be observed [32], giving rise to the formation of nanometric **MgO** organized in the form of a network of intercrystallite channels maintaining the original pseudo-hexagonal-platelet morphology of the precursor itself (Figure 3a,b).

At higher magnification, each channel appeared to be composed by an aggregation of monodispersed and roughly spherical **MgO** NPs having dimensions less than 10 nm (Figure 3c), also confirming the results coming from the XRD.

**Figure 4:**
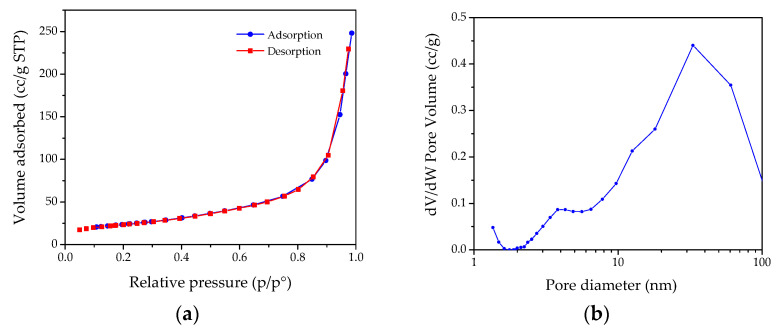
(**a**) Nitrogen adsorption–desorption isotherms for **MH** dry powders; (**b**) Barrett–Joyner–Halenda (BJH) pore size distribution curve determined by using the N_2_ desorption isotherm.

**Figure 5:**
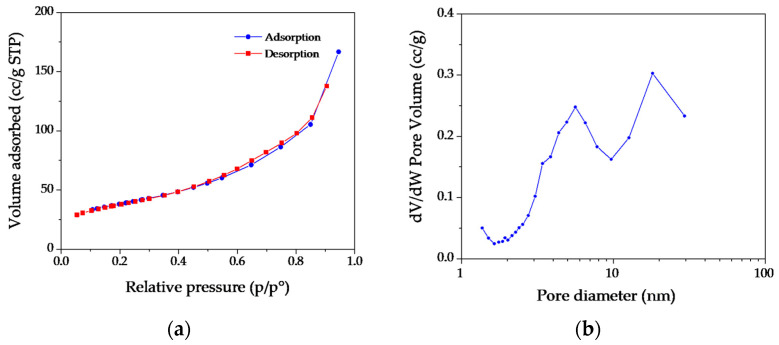
(**a**) Nitrogen adsorption–desorption isotherms for **MgO** dry powders; (**b**) Barrett–Joyner–Halenda (BJH) pore size distribution curve determined by using the N_2_ desorption isotherm.

Both for **MH** and **MgO** samples, the presence of small nanometric crystals organized to form each pseudo-hexagonal lamella is confirmed by BET surface area analyses, reported in Figure 4 and Figure 5, respectively. For the **MH** precursor, BET values up to 86 m^2^/g were recorded, which were values much higher than those reported in the literature [41,42,43]. Regarding the nitrogen desorption/adsorption measurements, the isotherms and the corresponding BJH (Barret–Joyner–Halenda) pore size distribution were reported in Figure 4. According to IUPAC classification, the adsorption isotherm was well matched to type IV, corresponding to the multilayer adsorption on micro and mesoporous solids [44]. At higher relative pressures, 0.90–1.0 p/p, vertical and parallel adsorption and desorption branches can be noted (Figure 4a), related to an H1 hysteresis loop and attributable to solids crossed by channels uniformly distributed in size and shape [45,46]. The results coming from the pore size distribution (Figure 4b) obtained by means of the BJH method showed that the **MH** dry powders are composed by pores mainly centred in the range 5–50 nm, confirming the mesoporous structure, probably deriving from the aggregation process of thin nanoplates.

Concerning the produced **MgO** powders, BET values up to 137 m^2^/g were obtained, while the N_2_ adsorption/desorption isotherm showed a type III characteristic, with an H3 hysteresis loop (Figure 5a). These results underlined the presence of large textural mesopores probably related to the **MgO** NPs’ aggregation [32], as also observed by the TEM technique. From the BJH analysis (Figure 5b), a bimodal distribution of mesopores in the range of 3–30 nm can be observed as well.

The cytotoxic activity of the **MgO** NPs towards cancer cells was assessed by performing tests on human melanoma cells. In particular, **MgO_50_**, **MgO_100_**, **MgO_200_**, **MgO_300_**, **MgO_400_** and **MgO_500_** suspensions, chosen for the treatments, were sonicated to reach a homogeneous distribution of the NPs and then added into the culture plates.

The same procedure was then repeated for human skin fibroblasts (HS27), to evaluate the influence of the treatment on healthy cells.

**Figure 6:**
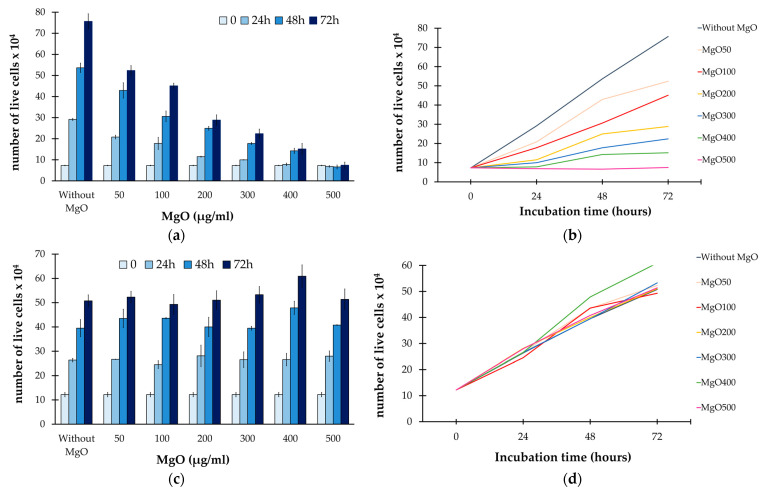
(**a**,**c**) Histograms representing the number of live melanoma and healthy cells in relation to the MgO suspension concentration as well as to the incubation time; (**b**,**d**) growth curves of melanoma cells and skin fibroblasts expressed as a function of the incubation time and the MgO suspension concentration too.

After 24, 48 and 72 h from the beginning of the treatment, a viable count was carried out, and the number of live cells of both melanoma and HS27 cells was shown (Table 2). Moreover, by using the results summarized in Table 2, the growth curves of melanoma cells and skin fibroblasts were determined as well (Figure 6).

From the obtained results, it was evident as without the addition of **MgO** NPs, the melanoma cells proliferated up to an order of magnitude by increasing the incubation time, with cell values ranging from 73,000 (t = 0) to more than 756,500 (t = 72 h).

Differently, when the **MgO** NPs were added, at already 24 h a clear cytotoxic effect was detected, showing a significant reduction in melanoma cell proliferation in a **MgO**-concentration-dependent manner (Figure 6a,b). In particular, after 3 days of incubation time, the culture plates treated with **MgO_400_** revealed a reduction in the cellular activity up to an order of magnitude, with a melanoma live cell counting ranging from 756,500 to 74,600 (see Table 2).

When the highest MgO concentration was reached (that is **MgO_500_**), a total inhibition of the cell growth was observed, with the number of detected live cells almost constant during the whole incubation time.

Regarding the skin fibroblasts (HS27), it was evident that as the MgO NPs, even at the highest suspension concentration, did not alter at all either the vitality or proliferation of healthy cells (Figure 6c,d).

Regarding the dead cells, identified by means of the microscope technique due to a dye solution containing Trypan Blue, mainly after 48 h of treatment an increase in mortality rate can be observed. The results were particularly marked for the treatment performed with the **MgO** suspension concentrations, showing an increase up to an order of magnitude in the mortality rate. In particular, the values ranged from 1.4% for the untreated sample to about 5% for samples treated with **MgO_200_** and up to 20% for the sample treated with **MgO_400_**.

Finally, the 50% inhibitory concentration (IC_50_) related to melanoma cells was calculated for all the considered incubation times; the obtained results were equal to 140.5 ± 17.68, 152.5 ± 13.43 and 123.5 ± 0.71 μg/mL after 24, 48 and 72 h, respectively.

Considering that the obtained IC_50_ values are generally lower than those reported in the literature [2,47] and given that the lower the IC_50_ value is, the greater the effect of the compounds is [47], the **MgO** NPs under this study can be considered very effective in terms of the cytotoxicity and proliferation inhibition of cancer cells, which are also able to preserve the healthy cells.

## 4. Conclusions

The intrinsic features of metal oxide nanoparticles in terms of antioxidant action as well as cytotoxic behaviour towards cancer cells has become the current intriguing challenge, introducing a new criterion for the development of extensive applications of the NPs themselves in the medical field. Among all of them, magnesium oxide NPs have been established at the forefront of nanomedical research thanks to their interesting properties in terms of crystallinity, stability, absorptivity, large surface area, reactivity and biocompatibility.

Most of the current synthetic routes for the MgO NP production reported in the literature are not able to satisfy the global challenges in terms of sustainability, green approaches and scalability, being frequently characterized by drawbacks related to high temperature/pressure conditions, expensive or sophisticated apparatuses, long synthesis times and, especially, a low yield of NP production.

In this task, the possibility provided by our innovative and sustainable route, to obtain in only 15 min up to almost 10 kg/week of Mg(OH)_2_ NPs acting as a precursor, can constitute an important goal for the extensive application of the MgO NPs, particularly in the medical field where large amounts of product are required.

In the present study, we evaluated the cytotoxicity of MgO NPs obtained from the calcination of a Mg(OH)_2_ precursor synthesized starting from our time–energy saving and cyclic method towards the cancer formation and growth by means of in vitro tests.

In particular, the MgO NPs, if formed of dry powders and having BET values up to 137 m^2^/g, appeared organized as a network of intercrystallite channels, preserving the original pseudo-hexagonal-platelet morphology of the precursor and formed by an aggregation of monodispersed and roughly spherical NPs < 10 nm.

The produced MgO NPs, dispersed in a PBS solution giving rise to different MgO suspension concentrations, were then used to evaluate their cytotoxic behavior towards melanoma cells and their influence on healthy cells. The growth curves revealed that when the MgO NPs were added, a clear cytotoxic effect was already detected at low incubation times (24 h) and at low MgO suspension concentrations, giving rise to a non-negligible reduction in cellular proliferation. After 72 h of treatment, by increasing the MgO suspension concentration, a reduction in the cellular activity up to an order of magnitude was observed. When the **MgO_500_** concentration was reached, a total inhibition of the cell growth was detected, with a number of live cells almost constant during the whole incubation time.

Finally, the results coming from the skin fibroblasts (HS27) revealed that the MgO NPs did not alter in any way both the vitality and proliferation of healthy cells.

## Figures and Tables

**Table 1:** Crystalline size, evaluated by the Debye–Scherrer formula, of **MH** and **MgO** samples.

ICSD 98-008-9823			MH NPs			ICSD 98-017-0905			MgO NPs		
*a = 3.1430 Å* *c = 4.7670 Å*			a = 3.1478 Å c = 4.7819 Å			*a = 4.2270 Å* *c = 4.2270 Å*			a = 4.2227 Å c = 4.2227 Å		
*hkl*	*I (%)*	*2 Theta (°)*	I (%)	Dhkl (nm)	<Dhkl> average value (nm)	*hkl*	*I (%)*	*2 Theta (°)*	I (%)	Dhkl (nm)	<Dhkl> average value (nm)
*001*	*96.30*	*18.60*	96.20	12.61	14.27	*111*	*11.40*	*36.80*	11.47	7.53	8.37
*010*	*2.60*	*32.88*	5.15	12.95		*002*	*100*	*42.75*	100	7.67	
*011*	*100*	*38.04*	100	13.13		*022*	*45.00*	*62.05*	44.95	8.35	
*012*	*37.80*	*50.88*	37.86	13.76		*113*	*4.90*	*74.37*	4.94	9.01	
*110*	*27.80*	*58.70*	27.75	14.28		*222*	*10.80*	*78.29*	10.83	9.27	
*111*	*15.70*	*62.14*	15.72	14.55							
*103*	*15.30*	*68.30*	15.39	15.10							
*201*	*9.70*	*72.12*	9.68	15.50							
*202*	*6.50*	*81.35*	6.45	16.60							

**Table 2:** The average values (Σ) of melanoma cells and skin fibroblasts (HS27) measured at 24, 48 and 72 h of treatment with different **MgO** suspension concentrations. The standard deviation (σ) was reported as well.

		Number of Live Melanoma Cells						
Time (hours)		*Without* *MgO*	*MgO_50_*	*MgO_100_*	*MgO_200_*	*MgO_300_*	*MgO_400_*	*MgO_500_*
0	**Σ**	**7.3 × 10^4^**						
	σ	0.14						
24	**Σ**	**29.16 × 10^4^**	**20.79 × 10^4^**	**17.78 × 10^4^**	**11.50 × 10^4^**	**9.98 × 10^4^**	**7.81 × 10^4^**	**6.86 × 10^4^**
	σ	0.51	0.89	2.86	0.23	0.11	0.62	0.39
48	**Σ**	**53.64 × 10^4^**	**42.90 × 10^4^**	**30.60 × 10^4^**	**24.95 × 10^4^**	**17.75 × 10^4^**	**14.25 × 10^4^**	**6.63 × 10^4^**
	σ	2.21	3.68	2.54	0.93	0.49	1.20	0.92
72	**Σ**	**75.65 × 10^4^**	**52.36 × 10^4^**	**45.09 × 10^4^**	**28.85 × 10^4^**	**22.40 × 10^4^**	**15.15 × 10^4^**	**7.46 × 10^4^**
	σ	3.61	2.33	1.23	2.47	2.18	2.62	1.40
		**Number of live skin fibroblasts**						
Time (hours)		*Without* *MgO*	*MgO_50_*	*MgO_100_*	*MgO_200_*	*MgO_300_*	*MgO_400_*	*MgO_500_*
0	**Σ**	**12.2 × 10^4^**						
	σ	0.91						
24	**Σ**	**26.36 × 10^4^**	**26.67 × 10^4^**	**24.54 × 10^4^**	**28.11 × 10^4^**	**26.53 × 10^4^**	**26.57 × 10^4^**	**27.97 × 10^4^**
	σ	0.74	0.08	1.58	4.40	3.21	2.54	2.18
48	**Σ**	**39.50 × 10^4^**	**43.50 × 10^4^**	**43.60 × 10^4^**	**40.00 × 10^4^**	**39.47 × 10^4^**	**47.86 × 10^4^**	**40.77 × 10^4^**
	σ	3.53	3.82	0.28	3.96	0.81	2.74	0.16
72	**Σ**	**50.75 × 10^4^**	**52.30 × 10^4^**	**49.30 × 10^4^**	**51.03 × 10^4^**	**53.28 × 10^4^**	**60.90 × 10^4^**	**51.36 × 10^4^**
	σ	2.47	2.40	4.10	3.86	3.39	4.67	4.30

For each MgO suspension concentration, two tests were performed.

## Data Availability

Data are contained within the article.
